# Supplementary material for: Bayesian refinement of protein structures and ensembles against SAXS data using molecular dynamics
Source: PLoS Comput Biol. 2017 Oct 18;13(10):e1005800. doi: 10.1371/journal.pcbi.1005800 (PMC5662244; doi:10.1371/journal.pcbi.1005800)
Supplement: S1 Archive — (BZ2) [file pcbi.1005800.s011.tar.bz2 › gromacs_4.62_bayesian_saxs_refi/share/html/online/rtp.html]

rtp file format


## rtp file format

|  |  |
| --- | --- |
| Main Table of Contents | **VERSION 4.6** |
| GROMACS homepage | **Sat 19 Jan 2013** |

---

### Description

The rtp file extension stands for residue toplogy.
Such a file is needed by pdb2gmx
to make a GROMACS topology for a protein contained in a .pdb
file. The file contains the default interaction type for the 4 bonded
interactions and residue entries, which consist of atoms and
optionally bonds, angles dihedrals and impropers.
Parameters can be added to bonds, angles, dihedrals and impropers,
these parameters override the standard parameters
in the .itp files.
This should only be used in special cases.
Instead of parameters a string can be added for each bonded interaction,
the string is copied to the .top file,
this is used for the GROMOS96 forcefield.

pdb2gmx automatically generates all angles,
this means that the [angles] field is only
useful for overriding .itp parameters.

pdb2gmx automatically generates one proper
dihedral for every rotatable bond, preferably on heavy atoms.
When the [dihedrals] field is used, no other dihedrals will
be generated for the bonds corresponding to the specified dihedrals.
It is possible to put more than one dihedral on a rotatable bond.

pdb2gmx sets the number exclusions to 3, which
means that interactions between atoms connected by at most 3 bonds are
excluded. Pair interactions are generated for all pairs of atoms which are
seperated by 3 bonds (except pairs of hydrogens).
When more interactions need to be excluded, or some pair interactions should
not be generated, an [exclusions] field can be added, followed by
pairs of atom names on seperate lines. All non-bonded and pair interactions
between these atoms will be excluded.

A sample is included below.

```
[ bondedtypes ]  ; mandatory
; bonds  angles  dihedrals  impropers
     1       1          1          2  ; mandatory

[ GLY ]  ; mandatory

 [ atoms ]  ; mandatory 
; name  type  charge  chargegroup       
     N     N  -0.280     0
     H     H   0.280     0
    CA   CH2   0.000     1
     C     C   0.380     2
     O     O  -0.380     2

 [ bonds ]  ; optional
;atom1 atom2      b0      kb
     N     H
     N    CA
    CA     C
     C     O
    -C     N

 [ exclusions ]  ; optional
;atom1 atom2

 [ angles ]  ; optional
;atom1 atom2 atom3    th0    cth

 [ dihedrals ]  ; optional
;atom1 atom2 atom3 atom4   phi0     cp   mult

 [ impropers ]  ; optional
;atom1 atom2 atom3 atom4     q0     cq
     N    -C    CA     H
    -C   -CA     N    -O


[ ZN ]
 [ atoms ]
    ZN    ZN   2.000     0
```

---

http://www.gromacs.org
